# Silence Is Golden. Six Reasons Inhibiting the Spread of Third-Party Gossip

**DOI:** 10.3389/fpsyg.2019.01120

**Published:** 2019-05-13

**Authors:** Francesca Giardini, Rafael P. M. Wittek

**Affiliations:** Department of Sociology, University of Groningen, Groningen, Netherlands

**Keywords:** gossip, social networks, goal framing theory, cooperation, organizations

## Abstract

Most of the current literature on gossip describes gossipmongers as incessantly sharing evaluative and valuable information about an absent third party in teams, groups, communities, and organizations. However, potential gossipers can similarly decide not to share what they know, depending on the content, the context, or their relationship with the other actors in the gossip triad. We argue that understanding the reasons why people do *not* gossip may provide useful insights into individual motives, group dynamics, and collective behaviors. This theoretical contribution first critically surveys the existing gossip literature with the aim of highlighting the conditions under which people might refrain from sharing third party information. We then propose to apply Goal Framing theory as a way to bridge a theory of the micro-foundations of human behavior with an analytical model of the gossip triad that disentangles the various ways through which senders, receivers, and objects of gossip may be interrelated. From a goal framing perspective, most research on gossip illustrates the mechanisms in which the hedonic gratification derived from gossiping is reinforced by gain or normative goals. However, a normative or a gain goal frame can prevent the gossip monger from spreading the information, and we argue that depending on different configurations of frames and relations between actors the perceived costs of sending gossip may be far higher than much of the previous literature suggests.

“*There is only one thing in the world worse than being talked about, and that is not being talked about”*

-(Oscar Wilde)

## Introduction

Gossip is meanwhile considered as one of the most powerful reputational mechanisms safeguarding cooperation in human groups (Emler, 1990; Giardini and Wittek, 2019a). This is noteworthy, since up until a couple of decades ago, gossip as a topic of scientific inquiry has been ridiculed as marginal and insignificant at best, certainly when compared to serious and big societal challenges like inequality, inter-ethnic conflict, or economic development. But there are many good reasons to disagree with this view. The main argument comes down to gossiping being virtually costless to produce anywhere, by anyone, while at the same time being an extraordinarily effective sanctioning tool that either prevents one’s fellow group members to deviate from norms, or get them back in line in case they did (Gluckman, 1963; Ellickson, 1994). That gossip is “cheap” and therefore permeating almost all social situations has been reiterated time and again, though the evidence supporting this claim is scant, and it is mostly based on few and outdated field studies based on recorded real life conversations (Emler, 1994; Dunbar et al., 1997). Making use of an innovative event coding methodology that he had tailor-made specifically for the purpose of overhearing conversations in British pubs, Emler’s fieldwork (Emler, 1994) revealed that people spent about 60–70% of their conversations talking about third parties not present. Gossip became a really popular topic with Dunbar’s bestseller *Grooming, Gossip and the Evolution of Language* (Dunbar, 1997), which linked gossip to increased relative brain size in primates and to the evolution of language in humans. More recent studies echo these earlier findings. A national survey among 1,000 mobile phone users carried out by the Social Issues Research Center study in Britain (Fox, 2001) reports that a quarter of women and a third of men in the sample engage in “mobile gossip.”

The mounting evidence about the presumed ubiquity of gossip was soon backed by theoretical accounts, with authors from different disciplines trumping each other with adding new arguments to the growing list of reasons why gossiping is among the most effective and efficient practices of social control in society (for a recent multi-disciplinary overview of research on gossip, see Giardini and Wittek, 2019b). Evolutionary psychology has contributed to this view about the ubiquity of gossip by stressing the many functions it served in hunter-gatherer groups: negative gossip is an informal tool for social control (Enquist and Leimar, 1993), but it is also fundamental to collect information which has fitness-related value, in terms of control of resources, sexual activity, alliances and conflicts, and reliability of potential partners (Dunbar et al., 1997; McAndrew and Milenkovic, 2002; Kniffin and Wilson, 2005; De Backer et al., 2007; McAndrew, 2019). The fact that anthropologists have described the practices and rituals of gossip in many different places across the globe has also contributed to the perception of gossip as a human universal (Haviland, 1977; Arno, 1980; Brenneis, 1984; Brison, 1992; Stewart and Strathern, 2004; Besnier, 2009; Boehm, 2019).

The bottom line that emerges is that there are good reasons to exchange gossip in almost any social situation humans can possibly find themselves in, in addition to some people being inherently more inclined to vent third-party information than others (Nevo et al., 1993). But the surge of interest in gossip as an object of scientific study, the evidence about its ubiquity, and the claims about its importance in regulating social behavior resulted in a gross oversimplification of the decision to gossip. If one follows some existing accounts (Emler, 1990; Coleman, 1994; Dunbar, 2004), disclosing socially relevant knowledge about third parties is a largely unproblematic decision for both the sender and the receiver, an act that looks much like a reflex: it is spontaneous and effortless (Taylor, 1994) from the side of the sender, and uncontested, if not highly appreciated for its multiple benefits (that range from entertainment to bonding and social control), by the receiver.

Portraying gossip like this not only trivializes the very act of disclosing third-party information, it also places it outside the realm of deliberate individual decision making. This is certainly a mistake, especially when considering gossip as *sharing evaluative information about an absent third party that the sender would not have shared if the third party were present, and which, according to the sender, is valuable because it adds to the current knowledge of the receiver*. The benefit of adopting this definition is twofold: it allows us to exclude idle-talk, in which information is not valuable, and at the same time we can refer to both positive and negative gossip, from norm violations, to information about a promotion or an important achievement. The sharing of valuable and often negative information about an absent third party is not inconsequential. Even if a sender might prefer to avoid it simply because a piece of gossip can be considered uninteresting, or it might be known already, there are many more reasons for that. Many field studies acknowledged the presence of normative expectations according to which grievances should not be handled indirectly, but directly (Ellickson, 1994; Lindenberg et al., 2006). Also, in Western industrialized cultures, gossiping tends to be seen as inappropriate (Taylor, 1994), and notorious gossip mongers are often subject to contempt rather than hailed for their willingness to constantly share information about others (Farley et al., 2010; Farley, 2011). Islam considers backbiting as the 41st Greater Sin, and also the Bible contains many references in which gossipers are condemned. For example, Psalm 101:5 reads: “Whoever slanders his neighbor secretly I will destroy^1^”. Business firms may consider gossiping as detrimental, with some even actively trying to “ban” it (O’Callaghan and Hartigan, 2015), and business coaches recommending to implement anti-gossip policies in the workplace. However, there is no explanation of the fact that the remedial norm against gossiping tends to be violated systematically (Ayim, 1994). This also means that gossip receivers massively breach eventual promises to the sender, and they will become senders themselves (for a discussion on the ethics of gossiping, see Westacott, 2011). The vast majority of studies on gossip makes no references to the norms against it, nor do these studies problematize their violation.

Another default assumption of much gossip research is that sharing gossip requires the existence of a strong interpersonal trust relationship. Whereas personal bonds like friendship with the receiver were indeed found to facilitate sharing information, this does not mean that absent or only weak ties keep people from sharing information about a third party (Ellwardt et al., 2012b). Another frequently reiterated claim links dense interpersonal networks to a high incidence of gossiping. However, an early sociometric study on gossip, carried out in five organizations and five classes of a business school, found no significant relationship between an individual’s tendency to gossip and their embeddedness in closed triads of interpersonal trust relations (Wittek and Wielers, 1998).

What gossip research so far has *not* done is inquiring when, under which conditions, and why gossipers may deliberately refrain from negatively gossiping about others. One reason for this neglect may be the implicit assumption that our current models suffice, because the causal statements about the presence of gossip (e.g., the more social ties an individual has, the more likely it is that this individual gossips) by logical implication also comprise the explanation of its absence (the smaller an individual’s personal network, the less likely it is that the individual gossips a lot). But there is a big difference between not gossiping because one lacks the opportunity for engaging in it – e.g., because one has no access to potentially interesting third-party information or to those who might have it, or one has limited opportunities to share it with others – and having this opportunity to gossip, but deciding not to.

The present contribution argues that our current models are not equipped to explain why somebody who is in the possession of socially relevant information about someone decides not to disclose it. Far from being a riskless activity, gossip can have quite severe consequences (Michelson et al., 2010; Martinescu et al., 2014). This certainly holds for the objects of gossip – a well-placed negative remark by a powerful gossiper can destroy their reputation for good – but also for the broader group. How likely is it that potential senders tend to disregard such sometimes quite disruptive consequences, and spread incriminating news without thinking twice? Given their consequentiality, it is likely that there will be a sizeable number of situations in which the question whether or not to disclose socially relevant information will be subject to considerable reflection on the side of a potential gossip sender.

Explaining gossip requires to explain also why sometimes actions with high diagnostic value, like norm violations or unexpected behaviors, are observed but not shared, thus delaying the discovery of the transgression and benefitting the target. An example of this on a global scale is the Weinstein case, in which there were allegations and complaints known to many, but it took years before the gossip spread, i.e., his misdemeanors became publicly known. On a local scale, it happens quite often that only after a person leaves the organization the colleagues start chatting and discover that everybody had experienced or witnessed some form of unexpected behavior, but nobody wanted to gossip about the target. How can we explain the lack of gossip in cases in which expectations about what is appropriate are violated, there are different observers who belong to the same group and are able to inform each other?

If gossip is, in Dunbar’s words, “the core of human social relationships, indeed of society itself. Without gossip, there would be no society” (p. 100, 2004), then we need a better understanding of the reasons why people *do not* gossip, because this may provide useful insights into individual motives, group dynamics and collective behaviors.

The remainder of this article first sketches the contours of our analytical framework, introducing goal framing theory and applying it to the identification of the conditions and mechanisms favoring non-disclosure of socially relevant third-party information. The framework is subsequently illustrated drawing on findings from existing research and putting forward six reasons that might prevent a sender from sharing gossip with a receiver. The final section discusses the limitations and implications for future research on gossip.

## Silence is Golden: An Analytical Framework

Why might people refrain from negatively gossiping about others? What are the consequences of this on cooperative exchanges, team dynamics and performance? Building upon goal framing theory (Lindenberg, 1997), we argue that the decision to gossip or not depends on the different goals of the gossip sender and on the social and relational context.

Goal-framing theory is a general theory of human motives which has been applied to the analysis of norm conforming behavior, and to the conditions favoring it (Lindenberg and Steg, 2007). Goal framing theory posits that human decision making and behavior is goal directed, and that only one goal can be salient at any given moment. This is also the goal that will provide the dominant frame for action. There are three goal frames: in the hedonic goal frame, the salient goal is “to feel better right now”; in the gain goal frame, it is “to guard and improve one’s resources,” and in the normative goal frame the salient goal is “to act appropriately.” These three overarching frames are arranged in an *a priori* hierarchy of relative strength or salience, with the strongest being the hedonic goal, followed by the gain, and the weakest being the normative one. In Lindenberg’s words (Lindenberg, 1997, p. 317): “The cognitive vehicle for informal social control is framing exactly because it allows opportunity costs of conformity to vanish into the background, greatly lowering opportunistic tendencies if the frames are strong.”

Analyzed from a goal framing perspective, most research on gossip illustrates mechanisms in which the hedonic gratification derived from sharing valuable information about an absent third party is reinforced by gain or normative goals. However, the same theory can be successfully applied to explaining how the kind of interdependence between the actors in the gossip triad can increase the salience of the gain and the normative frames, thus overruling the hedonic satisfaction provided by gossip. Goal framing theory is not a theory of gossip in itself, but it can be fruitfully applied to disentangling the motivations behind gossip as a conscious and purposeful decision.

Gossiping as a social conversation is an instantly gratifying activity that satisfies many individual needs for stimulation, self-confidence, and personal bonding (Foster, 2004). Various scholars have emphasized the deeply and intrinsically gratifying nature of gossiping, pointing to its “fun” part, a natural reflex that often brings joy or a “warm glow” in those involved (Stirling, 1956). It is also tightly intertwined with a wide variety of emotions (Waddington and Fletcher, 2005; Martinescu et al., 2019). Discussing the ethics of gossiping, (Westacott, 2011) lists eight different “pleasures” experienced by those engaging in gossip: *schadenfreude*, *smugness*, *a feeling of power*, *titillation*, *catharsis*, *people are especially interesting topic of conversations*, *solving mysteries* and *learning is enjoyable*. Analogously, Gambetta (1994) suggested that gossip is a pleasure in itself, and he linked it to curiosity satisfaction and emotional complicity as two evolved mechanisms which would explain the pleasure of sharing gossip with others. For example, studies interpreting gossip as spontaneous, altruistic punishment of free riders violating fairness norms are rooted in the assumption that the act of punishing free riders is a deeply gratifying deed, in which hedonic and normative motivations are satisfied (Beersma and Van Kleef, 2012; Feinberg et al., 2012). Similarly, hedonic and gain motives drive Burt’s echo and bandwidth explanations of gossip, which he developed as part of his structural hole theory. The latter is rooted in the assumption that individuals are rational gain seekers (Burt, 2001). According to Dunbar (1997), gossip in humans is what grooming is to primates: its primary purpose is to establish and maintain alliances with other group members who might be important sources of support against potential future threats, in particular from others. The major mechanism through which such personal bonds of interpersonal trust are reinforced is that grooming and gossiping create a sense of mutual obligation. Grooming, in turn, is “extremely effective at releasing endorphins…The flood of opiates triggered by being groomed (and perhaps even by the act of grooming itself) generates a sense of relaxation (grooming lowers the heart rate, reduces signs of nervousness such as scratching, and can so relax the gromee that it may even fall asleep” (Dunbar, 2004, p. 101).

If gossiping indeed triggers the release of endorphins, it contributes to the realization of hedonic goals (Lindenberg and Steg, 2007). More specifically, given the a priori salience of a hedonic goal frame and the fact that gossip – due to the ease with which we can share third party information – provides many hedonic stimuli (in the sense of immediate satisfaction of needs for confirmation, bonding, belonging, etc.), it is likely that people tend to share third party information whenever this is possible. However, we know that this is not always true and we argue that gain and normative concerns may either reinforce or temper the salience of the hedonic goal frame.

While one frame is salient and present in the cognitive foreground, the other two overarching goals will remain still active but in the cognitive background. Their changing strength can affect the salience and stability of the goal frame in the foreground. Where background and foreground goals are aligned, the background goal reinforces or strengthens the foreground goal, thereby contributing to its salience and robustness. For example, you may want to share information about the inappropriate behavior of a team member with one of your closest colleagues, and this hedonic goal can be strengthened by a normative one. If the actors are part of an organization in which reporting others’ misbehaviors is encouraged and praised as a norm complying action, this normative goal in the background will reinforce the hedonic one in the foreground, thus increasing the likelihood of gossip. Conversely, in situations where background goals are at odds with the foreground goal, the increasing salience of the former goal weakens the latter one’s, which may eventually lead to a frame switch in which the most salient background goal replaces the foreground goal. For example, the normative goal of complying to the rule of not talking behind a colleague’s back, i.e., the foreground goal, may come under pressure to the degree that not sharing the information in question is likely to damage you. The larger the personal price to pay, the more likely it will be that the gain goal of preventing damage for yourself overrides the normative one prohibiting gossip against a team member.

These examples illustrate how gain, normative and hedonic frames may be related, and how the decision to gossip, or not, might be interpreted as the result of these three goals, either in conflict or in combination. We argue that the perceived costs of gossiping may be far higher than much of the previous literature suggests, and by focusing on the goal frames of the sender it is possible to identify six reasons for not gossiping. Which goal frame is salient in a given situation is highly context dependent, and previous research has pointed to a large variety of context conditions and their potential impact on goal frames. One category of such conditions that is of particular importance for the present study is the kind and degree of *(inter)dependence* connecting the members of a potential gossip triad (Wittek, 1999; Wittek et al., 2000). For analytical purposes, we look into the three dyads that compose a gossip triangle: sender-receiver, sender-object, and receiver-object. We treat these roles as being mutually exclusive, even if we acknowledge that in reality they overlap: receivers and targets are often senders, and senders and receivers can become targets as well.

A focal individual is said to be unilaterally dependent on another individual if the latter’s actions can positively or negatively affect conditions or opportunities important to the focal person, whereas the reverse does not hold (Kelley and Thibaut, 1978). Two individuals are mutually dependent (i.e., they are interdependent) if both can affect each other’s goal achievement (Molm, 1994). Dependence relations can have both functional-instrumental and/or cognitive-affective roots (Lindenberg, 1997). Functional (inter-)dependence (Wageman, 1995) is given where individuals need to rely on the delivery of each other’s resources or actions, for example in order to be able to properly carry out one’s own tasks. Cognitive-affective (inter-)dependence (Agnew et al., 1998), refers to the “collective representation of the self-in-relationship,” i.e., a situation involving close involvement with and relational commitment to a specific other person.

Types and degrees of mutual or unilateral dependence have been shown to be related to the (chronic) activation of specific goal frames. More specifically, cognitive-affective interdependence tends to be associated with a strong normative frame. It activates a range of rights and obligations that is common within solidarity relations, including strong interpersonal trust, helping, and refraining from actions that could harm the other (Lindenberg, 1997). For instance, in a department in which people have been working together for long time, and they respect and appreciate each other, a sender might refrain from negative gossiping about a team member because he does not want to harm the person. In contrast, unilateral functional dependence is likely to trigger a gain goal frame, in particular if the dependence is negative, meaning that the more powerful party is in the position to mainly inflict harm, rather than convey benefits.

## Six Reasons Against Gossip

Few studies have focused on the variation in the amount of gossip observed, and they explain it in terms of the differences among potential gossipers. Previous research has focused on the individual characteristics of the senders, showing that individuals are more likely to gossip about people of similar age and same sex (McAndrew and Milenkovic, 2002), but also that the tendency to gossip can be framed as a reliable individual difference variable that refers to how much individuals are prone to discuss others’ behaviors or traits (Nevo et al., 1993). Negative feelings and emotions can motivate the sender to share what she knows as a reaction to the frustration and unpleasantness of anger, sadness or disgust (Waddington and Fletcher, 2005; Grosser et al., 2012). Finally, individuals differ in their moral values and this also might affect the decision to spread evaluative information about someone. According to Fernandes et al. (2017) the decision to gossip depends on the link between the moral values that individuals endorse and the violations or endorsement of these values by others.

However, relational factors can be equally important in explaining these differences. The role of interdependencies among the three actors, and the way in which these affect the goal frame of a potential sender, might temper the hedonic goal and make either the gain goal frame or the normative goal frame salient^2^. Building on the analytical framework proposed by Giardini and Wittek (2019a), we are interested in understanding the sender’s decision to withhold socially relevant information on the basis of the goal frame activated by either functional or affective dependence on the other two actors in the triad. The nature of the (inter)dependence linking individuals in these dyads will contribute to the salience of gain or normative goal frames which, in turn, can override hedonic goals, and thereby temper the inclination to gossip. For a potential gossipmonger in a salient gain goal frame, the decision whether or not to spread information that is potentially damaging for a third party will be guided by the aim to improve one’s situation or prevent it from deteriorating. This involves material and immaterial benefits and costs alike. Hence, gossip is spread in order to realize some personal net gain, but also to avoid losses. For example, the decision to gossip can be guided by the gain goal to improve one’s own reputation at the expense of somebody else, or to increase one’s own opportunities for a challenging assignment by discrediting one’s most important competitor. Mitigating, avoiding or preventing potential net costs can be equally important motives in a salient gain goal frame. If spreading information may benefit the receiver of the gossip at the sender’s expense, the sender will refrain from gossiping. This would happen, for example, when the actors in a gossip triad aim to maximize their status (Wittek and Wielers, 1998).

The situation is different if the normative goal frame is salient for the sender, since this frame results in a discount of the personal costs that come with complying to a norm. When normative concerns are dominant, they may either inhibit or favor the spread of gossiping. For example, in many social contexts there are remedial norms against talking behind people’s back. Potential senders in a normative goal frame may actually comply to this norm, therefore both gain and normative goals can inhibit the release of a gossip.

In what follows we outline six propositions defining how, respectively, salient gain and normative goal frames may induce a potential sender to refrain from gossiping on the basis of which of the three relationships in the gossip triad is considered. Table 1 provides a summary overview of the six possible combinations. We refer to the three conditions in which a salient gain goal frame inhibits gossiping, as, respectively, competition, deterrence, and externality. Conversely, when a salient normative frame hampers gossip, we term them as signaling, solidarity, and coalition conditions.

**Table 1:** Six gossip inhibiting conditions based on Goal Framing Theory.

	Functional interdependence	Affective interdependence
Sender-Receiver	*Competition*	*Signaling*
	Strong negative functional interdependence between potential gossip senders and receivers is likely to increase the salience of gain goal frame.	Strong cognitive-affective interdependence between potential gossip sender and receivers is likely to increase the salience of remedial norms against gossip.
Sender-Object	*Deterrence*	*Solidarity*
	The stronger a potential gossiper’s functional dependence on the object of gossip, the more salient the goal to avoid potential losses resulting from the object’s retaliation.	Strong cognitive-affective interdependence between potential gossip senders and objects is likely to increase the salience of solidarity norms proscribing to harm each other.
Receiver-Object	*Externality*	*Coalition*
	Strong functional interdependence between potential gossip receivers, objects, and the sender increases the likelihood that the spread of third-party information has negative externalities for the sender.	Strong cognitive-affective interdependence between potential gossip receivers and objects is likely to increase (a) the likelihood that the receiver will reveal the sender’s identity to the object, (b) the damage that a gossip sender can inflict on their relationship by sharing negative information about one of them.

**FIGURE 1:**
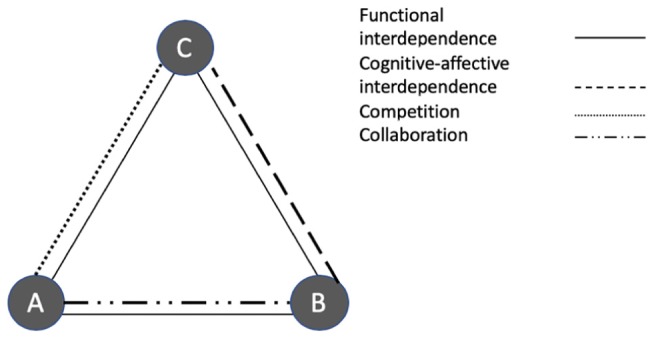
Visual illustration of the interdependences and relationships between the three actors in the example.

As an illustration, let’s consider the following example. It describes the various interdependencies connecting four members of a hypothetical team in a financial services company. Figure 1 provides a graphical summary of their interconnections. Chiara, Anthony and Bianca are colleagues working in the same department of a large company. Bianca is the most experienced of the three, Chiara just joined 3 months ago, whereas Anthony was hired a year ago. With both of them being new hires, they both are still in the probation period. All three of them have the same formal position, and report to the same supervisor. The work environment is competitive and a considerable part of their salary is tied to performance, as are promotion chances. There is also a yearly bonus, which can be allocated only to one of them. In addition to a bonus for individual performance, all members of the department receive a bonus if the total sales exceed a certain threshold. Anthony, Bianca and Chiara compete within the same client pool for the acquisition of new projects, and success in acquiring new clients is an important part of the performance. Bianca and Anthony have been assigned to jointly work on a large project for one of the company’s most important clients. This project requires close collaboration, frequent information exchange. Their professional expertise differs, and they need each other to complete the project. Bianca and Chiara know each other from school and are friends.

### Competition

The first mechanism, *competition*, can be illustrated by the *negative functional interdependence* and the resulting salient gain frame as it characterizes the relationship between Anthony and Chiara in our vignette. We argue that, since both are still on probation and are under a lot of pressure to acquire new projects, it is unlikely that they will share gossip with each other about Bianca if one of them happens to have information that may provide them with a personal advantage (e.g., knowing about Bianca not being able to solve a problem with a specific client, which may require reallocation of the client to somebody else in the team).

Both task and outcome interdependence (Kelley and Thibaut, 1978) can create social dilemma situations if they involve a competitive payoff structure. In such cases, they induce strategic behavior, also with regard to disclosing third party information. For example, private information about Bianca’s actions may provide competitive advantages for the person holding this information, being it either Chiara or Anthony. Getting first-hand knowledge about a project leader messing up with a project, with the likely consequence that he or she will be replaced as the project leader, may be valuable if I and another colleague are among the potential replacements. Sharing this information with her may increase my colleague’s opportunities (e.g., proactively manipulating the boss) at the expense of my own. Hence, in this case of negative outcome interdependence, the gain goal of the sender will be active, and he will have an incentive not to share information about problematic behavior of others. This is probably reinforced by another aspect of strategic situations: receivers aware of the negative interdependence with the sender will have legitimate doubts about the veracity of the information they receive, given that they know that it is not in the best interest of the sender to share it.

Proposition 1 summarizes our argument about the link between a salient gain goal frame and strategic interdependence:

*Proposition 1 (Competition)*: Strong negative functional interdependence between potential gossip senders and receivers is likely to increase the salience of the gain goal frame. This will temper the inclination to spread negative third-party information that may provide receivers with a competitive advantage.

### Deterrence

The second mechanism, *deterrence*, describes a situation in which potential gossip senders refrain from gossiping because of their strong dependence on the object of gossip, and the salient gain frame that this dependence induces. In our vignette, this situation is illustrated by Chiara, who will be unlikely to gossip with Anthony (receiver) about Bianca (object), because she strongly depends on Bianca’s help and advice.

In such situations of formal or informal unilateral (power) dependence, actors in a weak power position like Chiara may refrain from gossiping about the more powerful actor, because spreading sensitive information may make them vulnerable twice: the receiver, Anthony, may share this information with the powerful person, Bianca, and in case this happens, the powerful person may sanction Chiara, for example by ceasing to help and give advice. More generally, in most gossip research, the third party is portrayed as a passive object over whom people talk. But third parties may be far more proactive in their attempts to prevent damages to their own reputation, and deterrence is one of the strategies that can be used to achieve this objective. Deterrence has been equated with the idea that gossip constitutes a social sanction (Gluckman, 1963; Kerr, 1999; Keltner et al., 2008; Giardini, 2012), and the fear of being gossiped about by group members has been found to increase contributions to the group and reduce free riding (Piazza and Bering, 2008; Beersma and Van Kleef, 2011). Third parties may therefore actively approach those who observed their behavior, and talk to these potential senders, asking or “convincing” them not to spread the gossip. The likelihood for this to work may further increase to the degree that the potential senders depend on the third party. The possibility for future retaliatory action from the third party may be sufficiently threatening to deter a potential sender from spreading the incriminating information to others.

Finally, in formal contexts where sender and object are functionally interdependent, a potential sender observing a third-party misbehaving may prefer not to gossip with another peer, but consider the option to communicate the infraction to a formal control agent, especially if the infraction is serious.

*Proposition 2 (Deterrence)*: The stronger a gossiper’s functional dependence on the object of gossip, the more salient the goal to avoid potential losses resulting from the object’s retaliation. This will temper the inclination to spread negative information about the third-party.

### Externality

Our third mechanism, *externality*, captures those cases in which a potential sender refrains from sharing gossip because of the expected damage that this might cause herself due to her interdependence with the receiver and/or object. For example, Chiara (potential gossip sender), is likely to refrain from sharing gossip with Bianca (receiver) about Anthony (object) or vice versa, because she knows about the importance of Anthony and Bianca’s joint project for the firm. Sharing gossip may trigger conflict between them, jeopardize the successful completion of the project, and therefore put the realization of the departmental bonus at risk.

The standard explanation of gossip associates it with social control: sharing information about third parties – their bad character, their uncooperative behavior in the past – also serves as a warning for the receiver to be careful when engaging with the object of gossip (Sommerfeld et al., 2008; Feinberg et al., 2014; Milinski, 2016). Implications can be that the receiver loses trust in and reduces interaction with the object. Where receiver and object are functionally interdependent (Lindenberg, 1997), like in work groups, this can have severe repercussions on the cooperation between the two. For a variety of reasons, this may not be in the interest of a potential sender. For example, I may refrain from gossiping about a third party if I have strong reasons to believe that spreading this information ultimately may have severe negative repercussions for the group and myself. There may be situations in which a specific information about a leader may inevitably force him or her to resign, which in turn may make a group vulnerable. Similarly, sharing information about third parties may also come with the chance of conflict escalation, which might not be in my or the group’s interest.

*Proposition 3 (Externality)*: Strong functional interdependence between potential gossip receivers, objects, and the sender increases the likelihood that the spread of third-party information has negative externalities for the sender. This will strengthen the salience of a gain goal frame, tempering the inclination to gossip.

### Signaling

The fourth mechanism, *signaling*, is rooted in cognitive-affective interdependence and a salient normative goal frame. It reflects situations like the following: Bianca will not gossip with Chiara about Anthony, because she is Chiara’s friend, and therefore cares about Chiara perceiving her as someone who acts appropriately and complies to the department norms of not talking behind people’s back.

A strong interpersonal bond between sender and receiver is usually invoked as one of the major conditions facilitating or even triggering the exchange of gossip. For example, intra-organizational social network studies in a Dutch childcare organization (Ellwardt et al., 2012a,b,c) showed that gossip ties were highly reciprocal, and tended to be supplemented by trust ties over time, indicating multiplex reciprocity. However, a strong personal bond may also be an obstacle to share gossip, because this would violate the usually widely shared remedial norm that prohibits “talking behind people’s back,” and which stresses that if you have a problem with someone’s behavior, you should sort this out directly, and bilaterally with the person in question (e.g., Ellickson, 1994; Wittek, 1999). Whereas the remedial norm may hold in general – i.e., independently of whether or not one has a close tie to someone – a strong bond to a receiver may make this norm particularly salient for another reason: the sender might avoid being perceived as a nasty or revengeful person who gossips about colleagues in a conflictual situation. In fact, gossip can be perceived as a form of indirect relational aggression (Hess and Hagen, 2006). And as studies of interpersonal conflict in organization show, this indirect aggression is seen to reflect specific individual value orientations (Jeuken et al., 2015). That is, disclosing information about a third party also says something about the sender –her values and views and, more generally, into what matters to her. Complying to a remedial norm of appropriate management of conflicts with third parties therefore is a way of signaling one’s integrity. Never talking negatively about third parties with one’s friends reflects my determination to comply to the remedial norm of not talking behind people’s back, and it may reassure my friends that I also won’t gossip about them (Farley, 2011). Conversely, if I would constantly share gossip, my friends may start to doubt my integrity, and start to wonder to what degree I am inclined to also gossip about them with others.

In addition to having an identity signaling effect, complying to the remedial norm prescribing gossiping may also be fuelled by concerns of being sanctioned for violating this remedial norm^3^. A drastic medieval example for this sanction threat is the *Scold’s Bridle*, a “mask” which was used as a punishment for “rude, clamorous women” who were accused of having engaged too much in gossiping and quarreling. First mentioned in 1567, this instrument of torture impeded its bearer to speak (Boose, 1991). Attached to it was a bell, which made it impossible to move in public without attracting the attention of bystanders, thereby further humiliating the victim. Contemporary sanctions may be less severe, but the disapproval coming from a gossip receiver and the damage that this may do to a gossiper’s identity may nevertheless be a strong motivator to not share gossip. Proposition 4 summarizes:

*Proposition 4 (Signaling):* Strong cognitive-affective interdependence between potential gossip sender and receivers is likely to increase the salience of remedial norms proscribing gossip. The threat of being sanctioned in case of its violation will temper the inclination to share negative third-party information.

### Solidarity

Strong cognitive-affective interdependence also governs the *solidarity* mechanism. In our example, it is unlikely that Chiara will gossip with Anthony about Bianca, because she is Bianca’s friend. She therefore cares about protecting Bianca’s reputation, and harming it would harm basic principles of friendship.

Being connected to the object through a strong personal bond therefore is another condition that may keep potential gossipmongers from sharing evaluative information about the object (Tassiello et al., 2018). Not hurting those with whom we have a solidarity relationship is a strong social norm.

A potential gossip sender may experience uncertainty about the degree to which the behavior of the third party qualifies as free riding or incompetence. Since these may be serious allegations that can do a lot of damage, the sender may refrain from sharing potentially wrong information, because the severity of the consequences for the object may also have negative repercussions for the sender. If an affective bond is present, like friendship for instance, not sharing harmful information about the target can be a way to protect her reputation and standing in the group, at least until further information about the situation is collected by the sender. The sender can as well abstain from gossiping because of the consequences for the work climate in the team. For example, if a colleague observed a team member behaving inappropriately (e.g., insulting a client), leaking this information may eventually result in other colleagues getting angry at this team member. Particularly in situations where threats to team cohesion may pose a risk for all involved, it may be in the interest of a potential gossip monger to keep gossip for herself.

*Proposition 5 (Solidarity):* Strong cognitive-affective interdependence between potential gossip senders and objects is likely to increase the salience of solidarity norms prescribing not to harm each other. This will temper the inclination to spread negative third-party information.

### Coalition

The sixth and last mechanism proposed here, *coalition*, captures configurations in which there is a strong cognitive-affective relation between potential receivers and objects, which will result in a salient normative goal frame governing their relationship. For example, Anthony will not gossip with Chiara about Bianca or vice versa, because Chiara and Bianca are friends. This may have negative repercussions for Anthony, for example straining his relationship with both of them.

Hence, potential gossip senders may refrain from badmouthing a third party if they assume that the potential receiver and the object of the gossip have a strong personal bond. Several reasons may contribute to this reluctance. First of all, the strong cognitive-affective interdependence between receiver and object makes it likely that solidarity norms will govern their relationship. This coalition puts the sender in a risky position. Since solidarity norms prescribe that sensitive information and potential threats should be shared among partners with a strong personal bond, there is a fair chance that the receiver will inform the object about the sender’s attempt to badmouth her in her absence. As a result, gossiping exposes the sender to the risk of disapproval from both the receiver and the object, causing them to rescind or loosen the relationship with the sender. This is what the theory of “triadic closure” would predict. In his influential article on “The strength of weak ties,” Granovetter (1977) posits that if a strong link exists between A and B, and between A and C, then also B and C should have a positive connection, in accordance with the principle of cognitive balance (Heider, 1958). However, if the sender and the object have a strained relationship and the receiver and the object a positive one, the receiver might develop a negative relationship with the sender as a way to achieve cognitive balance. This is simplified by the saying the “enemy of my friend is my enemy.”

Second, if also the sender has a personal bond to one or both others, this will strengthen the normative concern of not causing damage to the relationship of the other two.

*Proposition 6 (Coalition)*: Strong cognitive-affective interdependence between potential gossip receivers and objects is likely to increase (a) the likelihood that the receiver will reveal the sender’s identity to the object, (b) the damage that a gossip sender can inflict on their relationship by sharing negative information about one of them. This will temper the inclination to spread negative third-party information.

## Discussion and Conclusion

People do gossip, and the literature on its motives and functions is already quite rich, but there is currently no explanation of the reasons why individuals who are in the position to spread valuable information refrain from doing so. Regardless of its consequences, but also of the differences among contexts, gossip researchers share the same assumption: gossip is effortless and therefore omnipresent. We know that both individuals and groups may greatly benefit from gossip as an inexpensive and indirect way of acquiring information through social comparison (Wert and Salovey, 2004), creating and strengthening social bonds (Dunbar, 1997), and learning group norms (Barkow, 1992). Gossip works also as a way of disciplining minor violations in groups (Giardini and Conte, 2012; Giardini et al., 2014), and in organizations it has been related both to positive outcomes, like bonding with colleagues (Rosnow and Fine, 1976), and negative consequences, like workplace bullying (Einarsen, 2000) and team disruption (Ribeiro and Blakeley, 1995). Although different, these studies share the same starting point: gossip is everywhere because it is cheap and effortless. Is it really the case? How to explain those situations in which people abstain from gossiping?

We answered this question by combining a structural theory of social behavior in which functional and cognitive interdependencies among the three actors in the triad are described as perceived and interpreted by the sender of the gossip. Gossiping is motivated by a hedonic goal, but it might be as well hampered by a gain goal or a normative goal. These three different frames can become more or less salient, depending on the contextual features, which here refer to the kind of tie between three different dyads (sender-receiver, sender-object, and receiver-object) and the kinds of norms present. We identify six conditions in which either the gain or the normative goal frame would prevent the senders from gossiping, depending on whether they would be more concerned with their own relationship with the receiver or the object, or with the relationship between the receiver and the object. We derived propositions for each combination, with the aim of translating our conceptual model into a set of testable hypotheses to be tested in future work.

Our paper contributes to the literature on gossip in three different ways. First, we complement the psychological literature on the motives for gossip by introducing goal framing theory and focusing on the hedonic goal as the main motivation behind gossip, and then articulating two complementary mechanisms, gain and normative frames, which could counterbalance it and then help understanding when gossip does not happen. Second, by focusing on the gossip triad we advance the current understanding of the dynamic and relational aspects of gossip. Network studies (for a review, see Ellwardt, 2019) show that dyadic and triadic relationships can explain gossip emergence and co-evolution. However, so far nobody has explicitly modeled the dyads-within-the-triad, and the varying effects that these ties can have on the gossiping itself. Third, in the organizational literature there is no conclusive evidence about the positive or negative effects of gossip on teamwork and performance (Beersma et al., 2019). We claim that looking at those situations when gossip does not occur might shed new light on team and organizations dynamics and results. For instance, the absence of gossip might be an indication of a lack of trust among colleagues, or of the presence of a conflict between them. Past research in organizational psychology, sociology and anthropology has stressed the importance of gossip in small groups of individuals who interact repeatedly, as in tribes, villages or teams within organizations (Kniffin and Sloan Wilson, 2010). However, the different degrees of interdependence among the actors are usually not explicitly defined, with the exception of power relationships. Kurland and Pelled (2000) distinguished between three kinds of power in the workplace, and they singled out the relationships between gossip, positive or negative, and its effects on the power of individuals who initiate it. They mention “gossiper-recipient relationship quality” but they do not include the target in their analysis, thus missing out on the triadic nature of gossip.

Our theoretical framework can also have interesting implications at the societal level. For instance, in the Weinstein case only after the scandal broke out and the legal actions became public people acknowledged having heard “the gossip” about it, but not having acted upon it or spread it. According to our theory, there are different explanations for this. The deterrence effect could be easily related to Harvey Weinstein’s powerful position in the movie industry, but also to his general wealth and connections. Also, an externality effect can be hypothesized if the sender was unsure about the kind of bond between the object (Weinstein in this case) and the receiver. It is equally likely that in an environment depicted as very competitive as the Hollywood industry, negative gossip about a successful producer can offer an advantage to competitors and detractors, thus indirectly hampering the sender. In this specific example, the normative goal frame might be generally less salient, but we can speculate on the likelihood of a sanction threat effect and on the coalition effect.

There are many questions still open, though. If the debate is to be moved forward, a better understanding of goal framing theory and its comparison with alternative theories of human motivation and behavior needs to be developed. The proposed framework seems very suited to an organizational context, in which interdependencies among individuals can be clearly spelled out. The presence of a formal structure, with tasks and functional interdependencies makes gains and norms visible and easy to identify and reason upon. The same does not necessarily apply to other kinds of collectives, like for instance communities or small-scale societies in which family relationships have a direct effect on the amount and kind of gossip. However, there are many organizational forms, which differ in several respects, and this study does not take these differences into account. This would be a fruitful area for further work. A related issue that was not addressed in this study was the role of actors’ embeddedness in the larger social structure. We limited our analysis to those within the triad, but of course they are part of a larger social structure which partially determines their opportunities, desires and intentions.

This research has thrown up many questions in need of further investigation. This conceptual framework was designed to understand when negative gossip does not occur, but we do not know whether it applies to positive gossip, too. Positive information about an absent third party is also common in organizations, and future work is surely needed in order to find out whether there are different mechanisms in place and to what extent they differ.

The issue of the effects inhibiting gossip is an intriguing one which could be usefully explored in further research. Contrary to what (Goodman and Ben-Ze’ev, 1994) wrote in their Section “Introduction” to an edited multi-disciplinary volume aptly called *Good Gossip*, “Gossip is proscribed in principle and generally frowned upon, but at the same time it is honored in day-to-day practice” (p. 1), we believe that there is much more to be gained by understanding when and why gossip does not happen in day-to-day practice, than by only focusing on the situations in which it happens.

## Author Contributions

FG and RW developed the theoretical framework and wrote the manuscript.

## Conflict of Interest Statement

The authors declare that the research was conducted in the absence of any commercial or financial relationships that could be construed as a potential conflict of interest.

## Footnotes

1 https://www.openbible.info/topics/gossipers

2 The salience of one or the other goal frame may have structural and situational reasons. Since the present study focusses on the consequences of goal frames for gossip, we do not further discuss the antecedents of goal frames.

3 This is an example for a situation in which a gain goal in the cognitive background supports the salient normative goal frame.
